# Synanthropic spiders, including the global invasive noble false widow *Steatoda nobilis*, are reservoirs for medically important and antibiotic resistant bacteria

**DOI:** 10.1038/s41598-020-77839-9

**Published:** 2020-12-01

**Authors:** John P. Dunbar, Neyaz A. Khan, Cathy L. Abberton, Pearce Brosnan, Jennifer Murphy, Sam Afoullouss, Vincent O’Flaherty, Michel M. Dugon, Aoife Boyd

**Affiliations:** 1 Venom Systems & Proteomics Lab, School of Natural Sciences, Ryan Institute, National University of Ireland Galway, Galway, Ireland; 2 Discipline of Microbiology, School of Natural Sciences and Ryan Institute, National University of Ireland Galway, Galway, Ireland; 3 Westway Health Ltd., Unit 120, Business Innovation Centre, National University of Ireland Galway, Galway, Ireland; 4 Marine Biodiscovery, School of Chemistry, School of Natural Sciences and Ryan Institute, National University of Ireland Galway, Galway, Ireland

**Keywords:** Microbial ecology, Clinical microbiology, Bacterial infection, Infection, Invasive species

## Abstract

The false widow spider *Steatoda nobilis* is associated with bites which develop bacterial infections that are sometimes unresponsive to antibiotics. These could be secondary infections derived from opportunistic bacteria on the skin or infections directly vectored by the spider. In this study, we investigated whether it is plausible for *S. nobilis* and other synanthropic European spiders to vector bacteria during a bite, by seeking to identify bacteria with pathogenic potential on the spiders. 11 genera of bacteria were identified through 16S rRNA sequencing from the body surfaces and chelicerae of *S. nobilis*, and two native spiders: *Amaurobius similis* and *Eratigena atrica*. Out of 22 bacterial species isolated from *S. nobilis*, 12 were related to human pathogenicity among which *Staphylococcus epidermidis*, *Kluyvera intermedia*, *Rothia mucilaginosa* and *Pseudomonas putida* are recognized as class 2 pathogens. The isolates varied in their antibiotic susceptibility: *Pseudomonas putida, Staphylococcus capitis* and *Staphylococcus edaphicus* showed the highest extent of resistance, to three antibiotics in total. On the other hand, all bacteria recovered from *S*. *nobilis* were susceptible to ciprofloxacin. Our study demonstrates that *S. nobilis* does carry opportunistic pathogenic bacteria on its body surfaces and chelicerae. Therefore, some post-bite infections could be the result of vector-borne bacterial zoonoses that may be antibiotic resistant.

## Introduction

Bacterial infections represent a major threat to human health. For example, typhoidal *Salmonella* causes 27 million annual cases of typhoid fever resulting in 223,000 deaths^1^, and non-typhoidal *Salmonella* is responsible for over 93 million cases of gastroenteritis leading to 155,000 annual deaths^2^. Bacterial infections contribute significantly to sepsis^3^ and in 2017, 49 million cases of sepsis resulted in 11 million deaths worldwide. Antibiotic resistance further increases the threat to human health with drug-resistant bacteria causing 700,000 annual deaths worldwide^4^. According to the World Health Organization’s (WHO) global action plan on antimicrobial resistance, it is essential that antibiotic resistance is tackled across every contact zone between humans and the environment^5^. Contamination of human dwellings, and more specifically food and water storage facilities, is a major issue^1^. As such, identifying the source of contamination is crucial for reducing the spread of pathogens.

Synanthropic animals (wildlife associated with human habitats) can be major reservoirs and vectors of pathogenic bacteria. Wild, domesticated and captive animals can be colonised by bacteria and act as reservoirs^6^, transmitting pathogens through physical contact, including bites, stings and scratches^7^. For example, rats have historically caused epidemics and rat-borne zoonotic pathogens are once again increasing across Europe^8^. However, some animal groups that can potentially spread pathogenic bacteria in and around human habitats are often overlooked. Recently, venomous snakes were identified as reservoirs for *Salmonella* with potential to contribute to the health crisis through shedding contaminated faeces around homes and vectoring bacteria during bites^1,9^. Moreover, a recent study demonstrated that bacteria can survive within the venom and venom glands of snakes and spiders^10^.

Spiders occupy a varied range of synanthropic niches^11^. They eat a diverse range of prey, with some capable of catching and consuming large arthropods, fish, lizards, snakes, birds, rodents^12–16^ and medically important pests, including mosquitos and house flies^17–19^. Some will readily feed on carrion^20–22^. Wild caught specimens of *Steatoda nobilis* were observed by us feeding on dead prey for up to eight days in laboratory conditions (unpublished data). The innate immune system of arthropods protects against pathogenic microbes^23–25^; however, once dead, the microbes are free to thrive and multiply on the corpse of their host. It is inevitable that spiders will encounter microbes through the environment or through feeding, especially on carrion. The potential therefore exists for spiders to harbour virulent bacteria^26,27^ and they have been implicated in bite cases that subsequently led to bacterial infections^28^.

The clinical manifestations arising from spider bites (araneism) are diverse^29–33^. For example, necrotic araneism (necrosis resulting from spider bite) is most commonly documented from bites by members of the *Loxosceles* genus, though infrequently other species are involved^26,30,34–37^. Bacterial infection following a spider bite could potentiate prolonged and debilitating pathologies^26^. Indeed, a study showed the presence of *Clostridium perfringens* in the venom and on the chelicerae of *Loxosceles intermedia.* When *C*. *perfringens* was conjugated with *L. intermedia* venom and injected into rabbits, their synergism increased the size of the dermonecrotic lesion^26^. This synergistic activity however has not yet been proven in humans^38^. The implication of the spider as the source of these bacterial infections remains controversial. Spiders generally avoid humans and bite only as a defensive response to being trapped^39^. The spiders are then often crushed, escape, or are captured using non-sterile methods, and as a result, comprehensive microbiological analysis is not possible. A previous study that identified bacteria on *Tegenaria agrestis* (hobo spiders) deemed them to be non-pathogenic^40^. The authors argue that infections associated with spider bites are typically caused by bacterial species commonly found in the environment and on human skin^38,40^. Moreover, spider venoms are considered a rich source of antibacterial peptides^41^ leading to the proposal that these are sterile environments that neutralise bacteria. In this scenario, infections are secondary to the spider bite itself^9,10,38,40,42^. We therefore face a conundrum in determining if infections are caused by opportunistic bacteria already present on the skin (secondary infections) or are vectored directly from the bite via the chelicerae (vector-borne bacterial zoonoses).

The noble false widow spider, *Steatoda nobilis,* has expanded its range across Europe^43^, (including Ireland^12,44,45^ and the UK^43^), through Western Asia^46,47^, and the Americas^43,48–53^. This species is increasingly linked to medically significant bites to humans, especially in Ireland and the UK^29,48,50,53,54^. As range expansion continues, so will the increase in bite cases^43^. Envenomation symptoms of *S. nobilis* bites include prolonged moderate to intense pain, swelling and erythema, piloerection, diaphoresis, facial flushing, feverishness, vasodilation of blood capillaries, and minor necrosis^29^. Two native species of spiders, *Amaurobius similis* and *Eratigena atrica*, are commonly found in and around houses and gardens throughout Europe. While both species are capable of biting humans, they are not a common source of complaint by the general public. Irish and British media regularly report on alleged bites by *S. nobilis* and a BBC report attributes one death to bacterial infection resulting from the bite^55^. In some media reports, the victims were said to be unresponsive to antibiotics, indicating a potential involvement of antibiotic resistant bacteria. However, media reports typically lack conclusive evidence of spider bites. This led Hambler to call for an urgent evaluation of the potential risk of bacterial transmission from bites by *S. nobilis*^55^. In an unpublished case series involving confirmed *S. nobilis* bites currently being assessed by the authors, three victims were treated for subsequent mild to debilitating bacterial infections, including cellulitis and dermatitis. One victim required hospitalisation and an aggressive course of intravenous antibiotics.

False widow spiders (genus *Steatoda*), like the closely related black widow spiders (genus *Latrodectus*), can occasionally subdue small vertebrates^13,16,27,56,57^ as they possess a fast-acting neurotoxic venom^13,58–60^. It is the presence of α-latrotoxin that can induce neuromuscular paralysis and death in humans following envenomation by *Latrodectus* species^59^. The venom protein composition of *S. nobilis* was recently characterised and revealed that approximately two-thirds of the venom is composed of *Latrodectus*-like toxins, including the most powerful toxin classes, i.e. α-latrotoxins, α-latroinsectotoxins, and δ-latrocrustotoxins^58^. Also present are the enzymes (e.g. metalloproteases, serine proteases, and chitinases) that are thought to cause tissue damage and thereby facilitate spread of venom toxins into the prey. In high concentrations, α-latrotoxin can cause localised cell death and, when potentiated by the presence of enzymes, induce necrosis^58^,thus providing substrate that could facilitate bacterial virulence^26^.

Previous studies on *Latrodectus hesperus* demonstrated the potential for spiders to vector bacteria during bite^27^. Chelicerae excised from 220 specimens recovered five pathogenic antibiotic resistant bacterial species. The microbial colonisers of *S. nobilis* chelicerae have never been investigated. Such a study would provide data to (1) explain why bacterial infections are increasingly associated with bites by *S. nobilis*, (2) explain why some patients are unresponsive to frontline antibiotics, and (3) determine if the etiological agent could be vectored directly from the spider’s chelicerae or transferred from the body surface on to the area of the bite site. This could have significant implications for advising first line medical staff who are treating bites by *S. nobilis* and help in choosing appropriate care and treatment.

The main objectives of this study were to (1) characterise the microbiome of the non-native *S. nobilis,* along with the native *A. similis* and *E. atrica*; (2) identify bacteria species residing on the body surface and chelicerae of the spiders and (3) test the susceptibility of these bacteria to antibiotics.

## Results

### Isolation and genus identification of bacteria on *Amaurobius similis*, *Eratigena atrica* and* Steatoda nobilis*

In the first stage of this study, we investigated the presence of bacteria on the bodies and chelicerae of 3 spider species. 9 bacteria genera were recovered from *A. similis*, *E. atrica* and *S. nobilis* (Table 1 and data not shown). 5 *Salmonella*, 1 *Bacillus*, 2 *Staphylococcus*, and 1 *Escherichia* species were recovered from 9 full body samples of *A. similis,* which included 3 *Salmonella* and a *Staphylococcus* sp. identified from bodies of euthanised spiders. *Salmonella* and *Bacillus* spp. were also identified on the body of *E. atrica* and a *Staphylococcus* sp. on the body of *S. nobilis*.

**Table 1:** Bacteria genera identified on chelicerae of *A*. *similis*, *E*. *atrica* and *S*. *nobilis*.

Spider Species^a^	Bacterial Genus	Occurrence^b^
*A. similis*	*Advenella*	1
*A. similis*	*Bacillus*	4
*A. similis*	*Pseudomonas*	1
*A. similis*	*Raoultella*	1
*A. similis*	*Salmonella*	1
*A. similis*	*Staphylococcus*	7
*E. atrica*	*Bacillus*	4
*E. atrica*	*Raoultella*	4
*E. atrica*	*Staphylococcus*	9
*E. atrica*	*Yersinia*	1
*S. nobilis*	*Bacillus*	3
*S. nobilis*	*Paenibacillus*	7

^a^34 chelicerae were tested from 3 spider species: *A. similis*—16; *E. atrica*—10; *S. nobilis*—8.

^b^Number of times the genus was isolated from a spider species.

**Figure 1:**
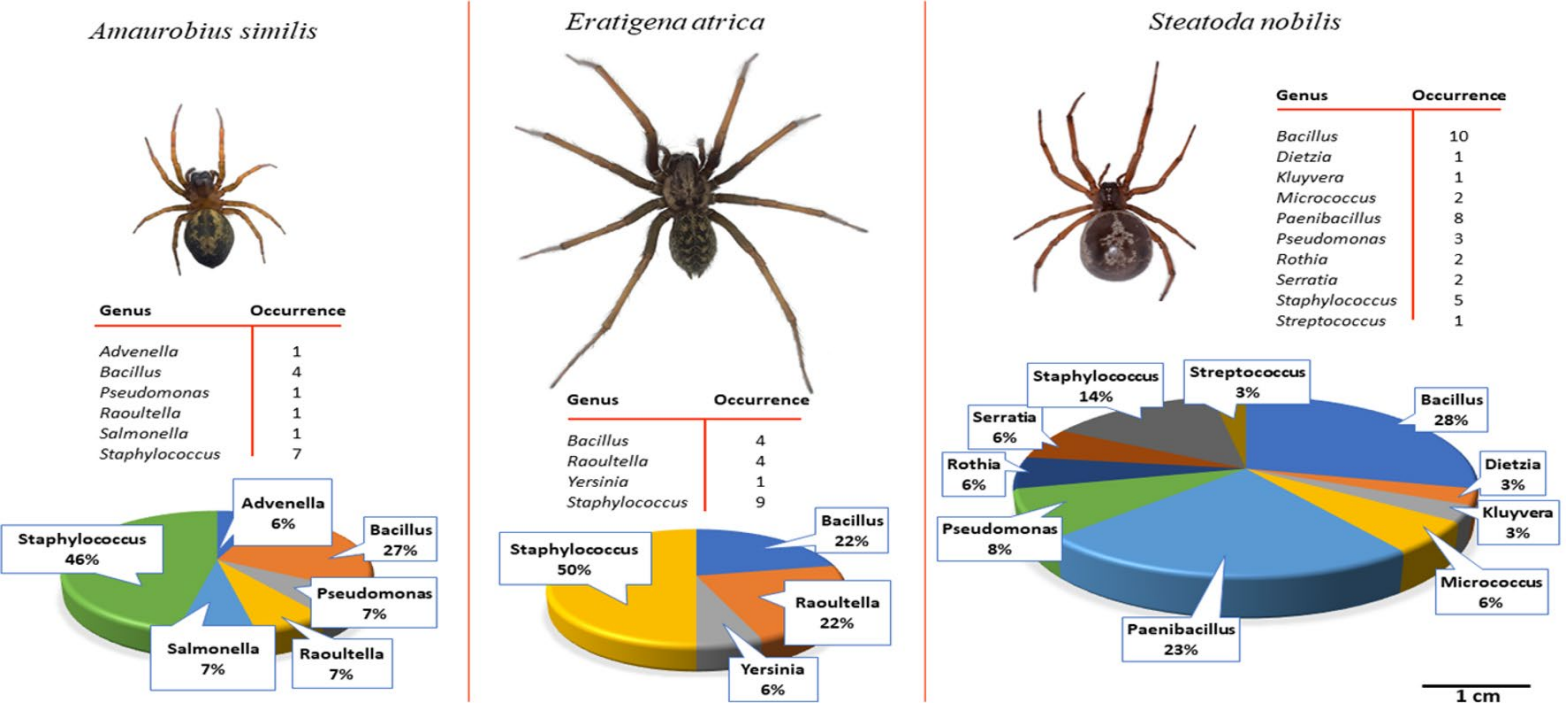
Comparison of the bacterial community composition and relative abundances from body and chelicerae surfaces of *A. similis*, *E. atrica* and *S. nobilis*. Photographs of the spider species are shown to scale. Data of chelicerae isolates are displayed for *A. similis* and *E. atrica*, and combined data of both chelicerae and body isolates are displayed for *S. nobilis*. The tables display the number of times isolates of each bacterial genus was isolated from a spider species. The pie chart displays the relative abundance of the bacteria genera isolated.

Of particular interest was the identification of 8 different bacteria genera on the chelicerae of these spiders (Table 1, Fig. 1). *Bacillus*, *Raoutella* and *Staphylococcus* spp, were recovered from the chelicerae of both *A. similis* and *E. atrica*, among which *Staphylococcus* spp. were predominant, occurring 7 and 9 times respectively, whereas *Paenibacillus* spp. were predominant on the chelicerae of *S. nobilis* being present in 7 out of 8 samples. The second most predominant genus was *Bacillus* which occurred in 4 samples from *A. similis* and *E. atrica* and in 3 samples from *S. nobilis*. *Pseudomonas* spp. were recovered from *A. similis* and *S. nobilis*, *Salmonella* and *Advenela* spp. each occurred once in *A. similis* and *Yersinia* occurred once in *E. atrica*.

### Isolation and species identification of bacteria in the *S. nobilis* microbiota

To test the hypothesis that spiders can carry pathogens and could play a role in infection following spider bites, bacteria were isolated from the body and chelicerae of *S. nobilis* and the sequence of the full-length 16S rRNA gene was determined to identify individual isolates to species level. *Streptococcus* and *Staphylococcus* were targeted by using selective CNA blood agar and Baird-Parker agar, respectively. Due to the increasing incidence of development in patients of infection associated with *S. nobilis* bites this species was an ideal candidate for this study. 20 chelicerae, 15 full body (5 of which had been dead for 24–48 h before sampling) and 2 “spider walk” samples of *S. nobilis* were analysed.

Twenty-five different bacterial isolates were cultured, and identified through 16S rRNA sequence analysis, within the microbiota of *S. nobilis*: 17 Gram-positive and 8 Gram-negative bacteria. For the majority of sequences, the percentage identity was > 99% with their respective most similar species (Table 2). Among these, 100% identity was found for 3 sequences to *Staphylococcus edaphicus*, *Staphylococcus warneri* and *Bacillus thuringiensis*. Two isolates displayed individual identity of 98% for *Bacillus pumilus* and *Streptococcus anginosus,* suggesting the isolates to be closely related to these two species.

**Table 2:** Bacteria isolated from *S. nobilis.*

Bacterial species	Source^a^	Growth on baird parker	Haemolytic	Pathogenic^b^
*Pseudomonas azotoformans*	C	−	−	−
*Pseudomonas peli*	C	−	−	−
*Rothia mucilaginosa*	C	−	−	+
*Staphylococcus capitis* (2)^c^	C	+	−	+
*Streptococcus sp.*^d^	C	−	−	+
*Bacillus aerius*	FB	+	+	−
*Bacillus altitudinis*	FB	+	+	−
*Bacillus licheniformis*	FB	+	−	+
*Bacillus mycoides* (1)	FB	+	−	−
*Bacillus mycoides* (2)	FB	+	+	−
*Bacillus thuringiensis*	FB	+	−	+
*Micrococcus endophyticus*	FB	−	−	−
*Bacillus sp.*^e^	FB-D	+	+	+
*Dietzia timorensis*	FB-D	+	−	+
*Micrococcus luteus*	FB-D	−	+	−
*Paenibacillus mobilis*	FB-D	−	−	−
*Pseudomonas putida*	FB-D	−	−	+
*Rothia amarae*	FB-D	+	−	−
*Serratia fonticola* (1)	FB-D	+	−	−
*Serratia fonticola* (2)	FB-D	+	−	−
*Staphylococcus capitis* (1)	FB-D	+	−	+
*Staphylococcus edaphicus*	FB-D	+	−	−
*Staphylococcus warneri*	FB-D	+	−	+
*Kluyvera intermedia*	SW	−	−	+
*Staphylococcus epidermidis*	SW	+	−	+

^a^37 samples tested from *S. nobilis*- 20 chelicerae, 15 full body (5 euthanised) and 2 spiders walk. *C* chelicerae; *FB* full body; *D* dead; *SW* spider walk.

^b^Pathogenicity was defined based on bacterial metadatabase BacDive (https://bacdive.dsmz.de/). “+” indicates bacterial species is associated with opportunistic infections due to underlying acute or chronic health conditions. “−” indicates no known association of bacterial species with infection.

^c^(1) & (2) indicate different strains of same species based on differing antibiotic susceptibility (Table 3).

^d^98% sequence identity to *S. anginosus.*

^e^98% sequence identity to *B. pumilis.*

Five isolates showed haemolytic activity on blood agar: 4 *Bacillus* spp. and 1 *Micrococcus* sp. Twelve isolates were related to human pathogenicity among which were 4 *Staphylococcus* spp., 3 *Bacillus* spp., and one each of *Rothia*, *Streptococcus*, *Dietzia*, *Pseudomonas* and *Kluyvera* spp. The association with human pathogenicity for each bacterial species was assessed using the bacterial metadatabase BacDive (Table 2).

Bacteria were isolated by each of the 3 sampling methodologies: 2 species were isolated from the agar plate with spider walks, 5 from the chelicerae and 18 from the full bodies (11 from dead spiders and 7 from live spiders). The 2 bacterial species from the spider walks (*Kluyvera intermedia* and *Staphylococcus epidermidis*) were different from the species found on other sites. The bacteria detected on the chelicerae were mostly distinct from the bacterial community on the full body, except for *Staphylococcus capitis* which was present on both sites. Differences in microbiota were also observed between bodies of live and dead spiders, with a wider variety of genera isolated from dead specimens, and primarily *Bacillus* spp. from live specimens.

### Anti-bacterial inactivity of *S. nobilis* venom

To investigate the hypothesis that bacteria can be transferred from the chelicerae into the host during the bite without being killed by the venom, *S. nobilis* venom was tested for its antibacterial property through Minimum Inhibitory Concentration (MIC) and agar diffusion assays. MIC assays were performed by testing diluted venom against pathogenic strains of *Escherichia coli*, Methicillin Resistant *Staphylococcus aureus* (MRSA) and *Listeria monocytogenes*. After incubation for 24 h in liquid media the absorbance at OD_590_ for growth of each pathogen in the presence of the highest concentration of venom (1:100) was 0.61 ± 0.09 (*E. coli*), 0.68 ± 0.06 (MRSA) and 0.34 ± 0.09 (*L. monocytogenes*), which was very similar to growth in the absence of venom (0.61 ± 0.01, 0.57 ± 0.06 and 0.35 ± 0.03, respectively). It was necessary for the venom to be diluted in the MIC assay due to limited venom availability. The agar diffusion assay was therefore deployed to assess the antibacterial activity of pure venom, as smaller volumes were sufficient with this method. Furthermore, we investigated the antibacterial ability of the venom against bacteria which are part of the spider microbiota. Two isolates recovered from *S. nobilis* chelicerae were the target bacteria in the assay: the Gram-negative *Pseudomonas azotoformans* and the Gram-positive *S. capitis*. 0.5 µl undiluted venom was applied to solid agar media spread with the bacteria. Alternatively, spiders bit the agar plate of bacteria directly. After 24 h no zone of bacterial clearance was observed on any of the culture plates (data not shown), indicating that the pure undiluted venom did not inhibit the growth of either species. These data demonstrate that the venom did not inhibit growth of either spider commensal bacteria or human pathogens, indicating that bacteria could survive in spider venom during transfer from the chelicerae to the host during a spider bite.

### Antibiotic susceptibility testing of strains isolated from *S. nobilis*

**Figure 2:**
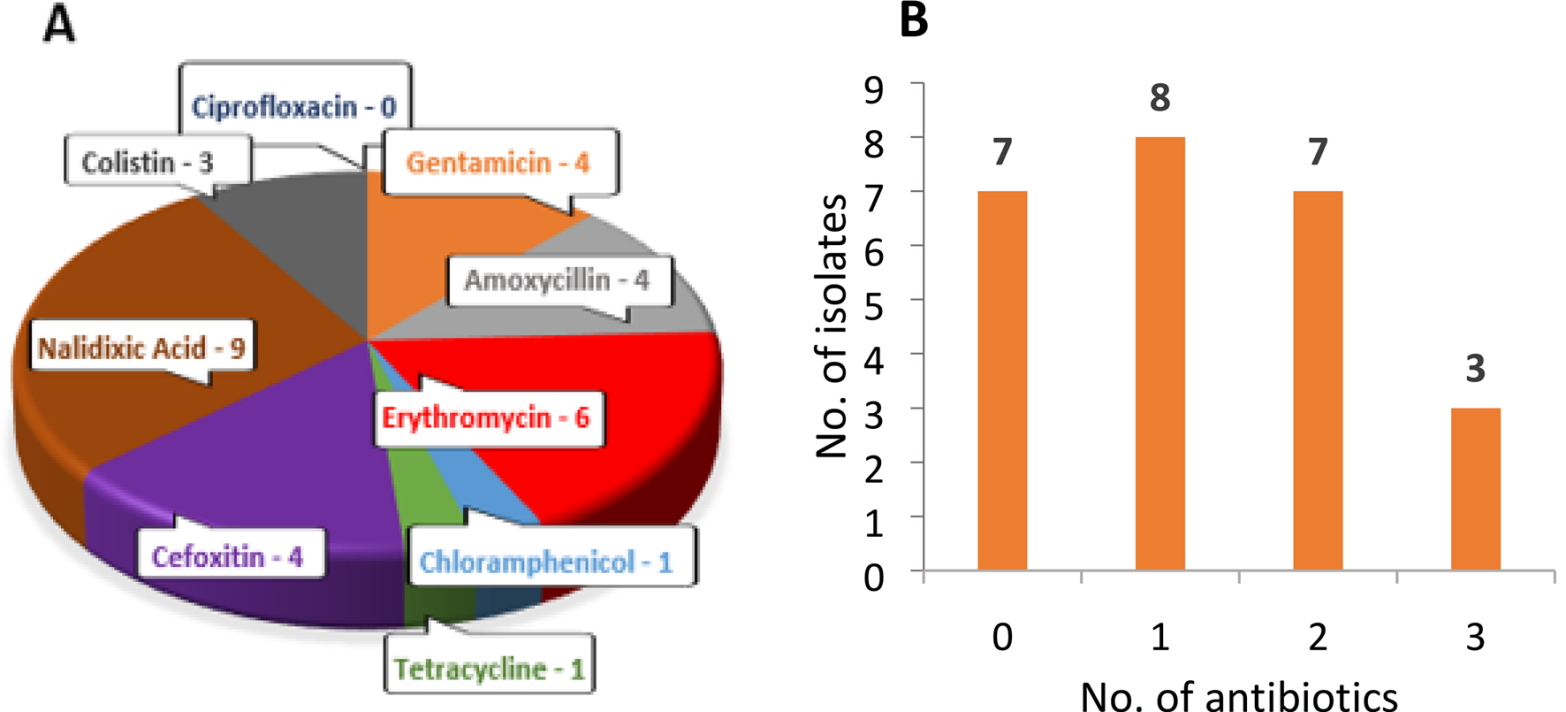
Antibiotic resistance profile of the bacterial community isolated from body and chelicerae of *S. nobilis*. (**A**) Number of bacterial isolates resistant to each antibiotic. (**B**) Number of isolates showing resistance to 0, 1, 2 and 3 different antibiotics.

Antibiotic susceptibility testing was performed by disk diffusion assays in accordance with the CLSI standards to determine the range of antibiotic resistance of the bacteria residing on *S. nobilis* and to determine which antibiotics would be the most effective in treating infection caused by those pathogens following a spider bite. The 25 isolates were tested against 9 antibiotics of 8 different classes, consisting of 8 broad spectrum antibiotics and 1 antibiotic with greater efficacy against Gram-negative bacteria (Colistin B) (Table 3). Of these 25 isolates, 10 are species listed in CLSI guidelines, namely—*Staphylococcus* (5), *Pseudomonas* (3), *Streptococcus* sp. and *K. intermedia*. For these isolates, resistance and susceptibility were inferred from their EUCAST breakpoints for each antibiotic. For the rest, lack of, or a minimal (≤ 8 mm), zone of clearance around the antibiotic disk was considered as resistant. Resistance to each antibiotic was displayed by at least one isolate, except for ciprofloxacin (Fig. 2, Table 3). Only one isolate was resistant to chloramphenicol (*S. edaphicus*) or tetracycline [*S. capitis* (2)], whereas 9 isolates resisted nalidixic acid and 6 were erythromycin resistant (Fig. 2, Table 3). 76% of isolates were resistant to at least one antibiotic and some isolates were multidrug-resistant. *Pseudomonas putida*, *S. capitis* (2) and *S. edaphicus* were notable for resistance to 3 antibiotics. All *Staphylococcus* isolates showed resistance to gentamicin and nalidixic acid, with the exception of *S. capitis* (1) for gentamicin and *Staphylococcus warneri* for nalidixic acid. *Dietzia timorensis*, *Rothia amarae* and the *Streptococcus* sp. isolate had identical resistance profiles with resistance to nalidixic acid and colistin only. These data demonstrate that there is a broad range of antibiotic resistance activity amongst bacteria residing on *S. nobilis* and the choice of antibiotic treatment for infected bites requires careful consideration.

**Table 3:**
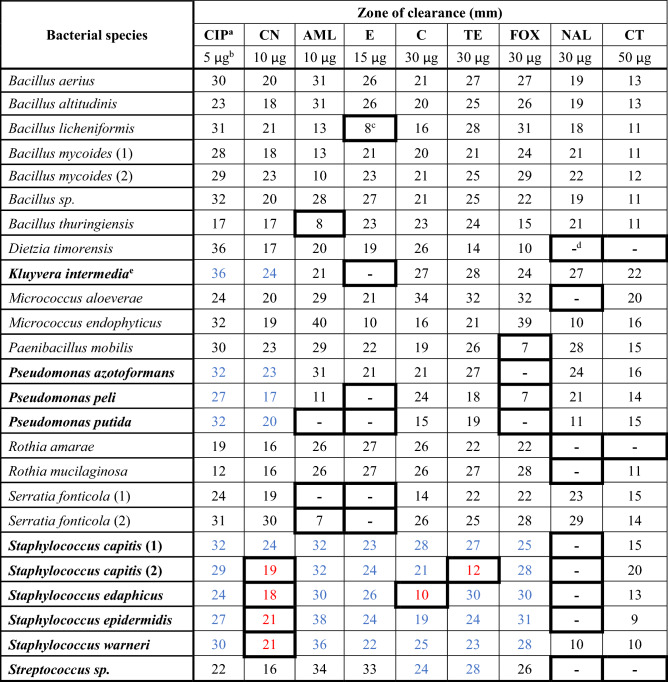
Antibiotic susceptibility of 25 bacterial isolates from *S. nobilis.*

Values shown are the average of three independent experiments performed in duplicate. SD for each value is ≤ 2 mm.

^a^Antibiotic abbreviations and classes: *CIP* ciprofloxacin (flouroquinolone), *CN* Gentamicin (aminoglycoside), *AML* amoxycillin (penicillin), *E* erythromycin (macrolide), *C* chloramphenicol, *TE* tetracycline, *FOX* cefoxitin (cephalosporin), *NAL* nalidixic acid (flouroquinolone), *CT* colistin (polymyxin).

^b^Amount of antibiotic in disk.

^c^Bold bordered boxes indicate resistance.

^d^-; no zone of clearance.

^e^Bolded species listed in CLSI guidelines.

Blue digits indicate susceptibility and red digits indicate resistance, according to CLSI guidelines; Black digits indicate antibiotic not recommended/applicable for respective species in CLSI guidelines.

## Discussion

The role of spiders in bacterial transmission has generated much debate^27,33,38,40^. In recent years, increasing media reports from Ireland and the UK^29,33,55^ claim that victims of the noble false widow spider *Steatoda nobilis* frequently suffer debilitating and sometimes fatal bacterial infections^55^. While these reports are largely unsubstantiated, there have been no studies carried out to validate the true risk of bacterial infections associated with this recently established spider.

In the first part of this study, the microbiomes from *S. nobilis* (8 chelicerae (C), 1 full body (FB)), *A. similis* (16 C, 6 FB) and *E. atrica* (10 C, 2 FB) were partially characterised*,* revealing diverse bacterial compositions of 9 different genera, most of which were detected on the chelicerae (Table 1). All these bacterial genera contain some species that are associated with human pathogenicity. Since *S. nobilis* is associated with bites that lead to infections, we focused the next part of this study on the bacteria present on body and chelicerae of *S. nobilis* (20 C, 15 FB, 2 spider walks (Table 2) and identified the bacteria to species level. In this subsequent investigation, 10 genera were recovered from body and chelicerae of *S. nobilis,* four in common with those found in the first part of this investigation. Testing the larger sample size of *S. nobilis* and identifying these isolates to species level allowed us to determine their potential for pathogenicity. The bacteria identified are members of microbiota of animals/humans and/or found in environmental settings. Eleven species are related to human pathogenicity (Table 2) and are recognised as opportunistic bacteria, among which *S. epidermidis*, *K. intermedia*, *R. mucilaginosa* and *P. putida* are designated as class 2 pathogens. We observed differences between bacterial communities on dead and living spiders. *Bacillus* spp. were abundant on living spiders, as found in a previous study^40^. However, for dead specimens only one *Bacillus* isolate was identified. The diversity of genera was greater on dead spiders and included *Dietzia* and *Serratia* spp. This may be explained by the occurrence of saprophytes, such as *S. fonticola, P. putida* and *M. luteus,* which thrive on corpses and could outcompete other bacterial species resulting in remodelling of the microbiota diversity and abundance^63^. Different bacterial communities were observed between sites of the living spiders. In contrast to full body sites, *Bacillus* spp. were not recovered from chelicerae or spider walks, indicating they probably reside more abundantly on body parts such as the abdomen. From the spider walk only *Kluyvera* and *Staphylococcus* spp. were isolated, possibly due to the low sample size. The chelicerae had the most diverse communities, including *Pseudomonas*, *Rothia*, *Streptococcus*, and *Staphylococcus* spp. This is possibly explained through direct exposure of the chelicerae to, and penetrating into, dead prey, in addition to contact with their legs/feet during grooming.

*Staphylococcus* spp. were recovered from *S. nobilis, A. similis* and *E. atrica,* of which four species recovered from *S. nobilis* were identified. Among them, *S. epidermidis* is a known human pathogen and responsible for severe illnesses, including bacteraemia, urinary tract infections, endocarditis, septicaemia and nosocomial sepsis originating from medical devices such as catheters and central lines^61^. Other *Staphylococcus* species identified can be opportunistic human pathogens, i.e. can cause severe infection in a host with a weakened immune system, an altered microbiota (such as a disrupted gut microbiota), or breached integumentary barriers, and are considered as typical components of the skin microbiome^64–66^.

*Pseudomonas* are ubiquitous in the environment and some species are associated with human infections. *Pseudomonas* spp. were recovered from *A. similis* and *S. nobilis* of which three species were identified, and of these one is related to human pathology. *P. putida* can cause bacteraemia, skin, soft tissue, and urinary tract infections, localised infections, pneumonia, peritonitis, septic arthritis, meningitis, and septicaemia^67,68^.

Two species of *Rothia* were recovered from *S. nobilis*, of which one is related to human pathology. *R. mucilaginosa* is a common constituent of the oral and upper respiratory microbiota. It is commonly associated with teeth and gum disease, but is now considered an emerging opportunistic pathogen, especially in immunocompromised patients associated with endocarditis, pneumonia, arthritis, meningitis, skin and soft-tissue infections, prosthetic joint infections, and endophthalmitis. For example, it was isolated from five cancer patients who developed bacteraemia^69,70^.

The native spiders *A. similis* and *E. atrica*, are common synanthropic spiders throughout Europe and neither species is considered to pose a threat to the general public. Bites are not thought to be common and therefore the risk of transmission resulting in infection is likely to be low^40^. Some bacteria genera from *T. agrestis* that were reported previously, were also identified in our study, e.g. *Bacillus*, *Paenibacillus*, *Pseudomonas* and *Staphylococcus*, and of those isolates identified to species level, *Bacillus thuringiensis* is present in both datasets. This species is now recognised as pathogenic, and our study shows it can display antibiotic resistance. In our study after allowing *S. nobilis* to walk on petri dishes with BHI agar, we recovered *K. intermedia* and *S. epidermidis*, indicating that spiders have potential to shed bacteria on the surfaces they touch. Following the rapid expansion of *T. agrestis* in North America, local media reported that the species was responsible for envenomation-led necrotic lesions. This claim has since been debunked, as with other spiders such as yellow sac spiders from the genus *Cheiracanthium*^30^. However, the claims that *S. nobilis* may be involved in severe envenomation events in Ireland and the UK appear to have some merit. As the geographical range of *S. nobilis* expands and their overall density increases in heavily urbanised areas, envenomations are becoming a more common occurrence. As a result, the transmission of pathogenic microbes during a bite event is now a cause for concern. The role of bacterial araneism is controversial; however, it is accepted that it is experimentally plausible for spiders to vector bacteria, and a confirmed infection vectored directly from a spider bite is discussed in the literature^28,38^. We demonstrated here that (1) a wide range of bacteria ubiquitous in the environment are carried on spider chelicerae and exoskeleton (Fig. 1), and (2) some are potentially pathogenic and involved in a wide range of clinical manifestations. In total, 11 species of potentially pathogenic bacteria were isolated from bodies or chelicerae of *S. nobilis*. We believe this clearly demonstrates the potential for bacteria to be vectored during bites and that it is just as likely that infections arise zoonotically as from commensal bacteria present on the skin (as is the current consensus)^9,10,38^.

In the case of *S. nobilis*, vectored infection may be facilitated by the venom’s ability to kill localised skin cells^58^, potentially disrupt normal immune response^30^, and provide substrate for bacteria to thrive. Moreover, *S. nobilis* typically bite humans when accidently trapped or squashed between the skin and clothing/bed sheets^29,54^. Therefore, the site around the bite could be contaminated by bacteria present on either the chelicerae or the body of the spider. Previous studies reveal spider venoms as rich sources of antibacterial peptides^41^ that could neutralise bacteria in paralyzed prey^38,71^. However, recent advances in venomics studies confirms that spider venoms are not sterile and should be viewed as microenvironments^9^. The results here demonstrate that *S. nobilis* venom has no inhibitory effect on bacterial growth, suggesting that the venom is unlikely to eliminate bacteria from the chelicerae.

Since the development of penicillin and subsequent antibiotics in the 1940s, there has been a rise in antibiotic resistant bacteria^72^ which currently kill over 700,000 people annually ^4^. Therefore, it is important to determine how antibiotic resistant bacteria move through the environment and establish contact zones between humans and the environment^5^. Pathogenic bacteria recovered from the chelicerae of black widow spiders^27^ included multiple antibiotic resistant strains, with fluoroquinolones and aminoglycosides recommended as the most efficient antibiotics for treating infections arising from black widow bites. Out of three confirmed bite cases by *S. nobilis* that resulted in dermatitis (data unpublished), one of the victims was unresponsive to antibiotic treatment. We tested the susceptibility of 25 bacteria recovered from *S. nobilis* against nine antibiotics used by front line medical staff and 19 antibiotic-resistant strains were identified (Table 3). The most resistant isolates were *P. putida*, which showed resistance to three broad range antibiotics (amoxicillin, erythromycin and cefoxitin), *S. capitis* (2) which also showed resistance to three completely different class of antibiotics (gentamicin, tetracycline and nalidixic acid) and *S. edaphicus* which showed resistance to gentamicin, chloramphenicol and nalidixic acid. *S. capitis* and *S. edaphicus* are the only isolates in this study to show resistance against tetracycline and chloramphenicol, respectively. In terms of resistance shown by the recovered isolates to each antibiotic (Fig. 2A), 9 of the isolates showed resistance to nalidixic acid followed by erythromycin (6), cefoxitin (5), gentamicin and amoxycillin (4), colistin (3), tetracycline (1) and chloramphenicol (1). All bacteria recovered from *S. nobilis* were susceptible to ciprofloxacin. An abundance of multidrug-resistant isolates were identified with 3 isolates resistant to 3 different antibiotics and 7 isolates resistant to 2 antibiotics (Fig. 2B). These data support the fundamental need to identify bacteria from spider bite victims. Additionally, there is a need for catalogues of the microbiota of spiders and cross-reference databanks with pathogenicity and antibiotic-resistance to better inform appropriate treatment for infections associated with spider bites.

## Conclusion

Our study demonstrates that the non-native *S. nobilis* and two native spider species, *A. similis* and *E. atrica*, carry opportunistic pathogenic bacteria on their body surfaces and chelicerae. Bacteria may be vectored directly from the spider, and as a result, post-bite infections may be the result of vector-borne bacterial zoonoses. Some of the bacteria carried by spiders are multidrug-resistant. Furthermore, our results showed that the venom of *S. nobilis* has no inhibitory effects against bacterial growth, indicating that it is most likely not a barrier to bacterial infection resulting from a spider bite. We believe this study provides a baseline for future research targeting synanthropic spider species to determine bacterial compositions and develop a database of bacterial species isolated from spiders, and to determine links to human disease.

## Methods

### Spider and venom collection

Specimens of *Amaurobius similis, Eratigena atrica,* and *Steatoda nobilis* were collected in Ireland, from garden walls and park railings in Lucan, Co. Dublin, Edgeworthstown, Co. Longford, Galway city, Co. Galway and Ferrybank, Co Waterford. Specimens were collected using sterile forceps, placed immediately into sterile tubes, and transported to the lab. Species identities were confirmed using identification guides specific to *S. nobilis*^12^ and Collins Field Guide for all other spiders^62^.

Using aseptic techniques, the specimens were dispatched, and the chelicerae were either clipped or swabbed. For whole body cultures, spiders were either submerged in media or swabbed. For surface colonisation analysis, spiders walked directly on Brain Heart Infusion (BHI) agar. The most common method for euthanising arthropods is dispatchment. A select number of spiders were euthanised using CO_2_, and immediately processed to determine if bacteria was recoverable by this alternate method.

For venom extractions, *S. nobilis* specimens were anesthetized using CO_2_ for 2 min and venom was extracted by electrostimulation with repeated pulses delivered at 15–20 V. Venom droplets were collected from the venom pores located on the outer subterminal part of the chelicerae using 5 µl microcapillary tubes modified with a tapered end for maximum efficiency. Venom from approximately 100 specimens was pooled and then flash-frozen in liquid nitrogen and stored at − 80 °C.

### Preliminary testing for microbiomes from *A. similis*, *E. atrica*, and* S. nobilis* and 16S rRNA gene amplification, sequencing, and analysis

Whole bodies or chelicerae from three species of spiders: *A. similis*, *E. atrica, and S. nobilis* were transferred into 750 μl (10% dilution) of Luria Bertani (LB) broth, Nutrient broth (NB), Tryptic Soy broth (TSB), MRS broth and BHI broth, and incubated at both 37 °C and 10 °C. Whole culture from each spider or chelicerae were pelleted, DNA was extracted collectively from each sample using the QIAGEN Dneasy Blood & Tissue Kit and V3-V4 region of 16S rRNA was amplified using 341F 5′-CCTACGGGAGGCAGCAG-3′^73^, and 806R 5′-GGACTACHVGGGTWTCTAAT-3′^74^. The amplified product was then sent to GATC Biotech for sanger sequencing. A BLAST search was carried out with the obtained sequence using the NCBI rRNA/ITS database (https://blast.ncbi.nlm.nih.gov/Blast.cgi).

### Bacterial isolation from *S. nobilis* and 16S rRNA gene amplification, sequencing and analysis

For isolating surface bacteria, *S. nobilis* spiders were washed individually with 5 ml BHI broth for 5 min. Some spiders were washed immediately after dispatchment, while others were processed 24–48 h after death. The wash media was then incubated at 37 °C overnight. For isolating bacteria from chelicerae, clipped chelicerae from each individual spider were inoculated into BHI broth and incubated at 37 °C. After 24 h incubation, the cultures were diluted and plated on BHI agar and incubated 48 h to 72 h at 37 °C. Selective media, Baird-Parker agar and TS-blood agar supplemented with colistin and nalidixic acid, were also inoculated with overnight cultures and incubated 48 h to 72 h at 37 °C. Colonies with different morphologies were selected for further analysis.

The 16S rRNA gene was amplified using *Taq* polymerase (Bioline) and universal primers, 27F (5′-AGAGTTTGATCATGGCTCAG-3′) and 1492R (5′-GGTTACCTTGTTACGACTT-3′) using Colony PCR^75^. The PCR product was purified using the Wizard SV Gel and PCR Clean-Up System (Promega) and sequenced using primers, 27F 1492R (Eurofins Genomics, Germany).

A BLAST search was carried out with the obtained sequence using the NCBI rRNA/ITS database (https://blast.ncbi.nlm.nih.gov/Blast.cgi). Closest bacterial species were identified using Blast tree view produced by Blast pairwise alignment.

### Inhibitory effects of *S. nobilis* venom against pathogens

Antibacterial activity of *S. nobilis* venom was assessed by agar diffusion assay and Minimum Inhibitory Concentration (MIC) assay. Agar diffusion assay was carried out against *S. capitis* (2) and *P. azotoformans* (isolated from *S. nobilis* chelicerae). Spiders were stimulated to aseptically bite Mueller–Hinton agar spread with 100 μl of adjusted overnight bacterial culture (0.8 OD_590_). We could observe the fangs penetrating the agar in a biting motion and also observe venom being expelled from the fangs. The restraining of the spider was enough to stimulate the bite and therefore no other manual stimulation was required. In addition, 0.5 μl neat venom was spotted onto the agar plate containing bacteria. Plates were incubated for 24 h at 37 °C and then assessed for zones of bacterial clearance.

The average volume of venom that each spider produces is approximately 0.22 µl (with a maximum of approximately 0.6 µl). Due to the limited amount of venom available, MICs were carried out by diluting the samples to achieve usable volumes. The MICs were performed against clinical isolates *E. coli* DSM10973, MRSA BH1CC and *L. monocytogenes* EGD-e. An overnight culture was adjusted with Muller-Hinton broth to an inoculum density of 1 × 10^6^ cfu ml^−1^. Starting with a 1:100 dilution of the venom, twofold serial dilutions of the venom, was tested against all the pathogens in a final inoculum of 5 × 10^5^ cfu ml^−1^. After 24 h incubation at 37 °C, absorbance at 590 nm was measured using a microplate reader (Tecan) with Magellan software.

### Antibiotic susceptibility testing

Disk diffusion assays were carried out to determine antibiotic susceptibility. Experiments were conducted according to the Clinical and Laboratory Standards Institute (CLSI) guidelines^76^. 6 mm disks preloaded with each antibiotic (Oxoid) were placed onto Mueller–Hinton agar plates that had been spread with 100 µl overnight bacterial culture (1 × 10^8^ cfu ml^−1^). Plates were incubated at 37 °C for 18 h and the clear zone around each disk was measured using a ruler and interpreted according to European Committee on Antimicrobial Susceptibility Testing (EUCAST) breakpoints^77^ Bacterial spread plates without antibiotic disks were used as negative control and the bacteria grew as a lawn each time. Three independent experiments were performed in duplicate.

### Ethical statement

No ethical approval was required to work with spiders. The three bite victims have provided the authors with written consent to use their case history and other relevant details to produce manuscripts intended for publication in scientific journals. They are aware that such publications may be available to the public both in print and on the Internet.

## Data Availability

The datasets generated and analysed during the current study are available from the corresponding author on reasonable request.
